# Structural and catalytic insights into HoLaMa, a derivative of Klenow DNA polymerase lacking the proofreading domain

**DOI:** 10.1371/journal.pone.0215411

**Published:** 2019-04-10

**Authors:** Michael Kovermann, Alessandra Stefan, Anna Castaldo, Sara Caramia, Alejandro Hochkoeppler

**Affiliations:** 1 Department of Chemistry, University of Konstanz, Universitätstraße, Konstanz, Germany; 2 Department of Pharmacy and Biotechnology, University of Bologna, Bologna, Italy; 3 CSGI, University of Firenze, Sesto Fiorentino (Firenze), Italy; Istituto di Genetica Molecolare, ITALY

## Abstract

We report here on the stability and catalytic properties of the HoLaMa DNA polymerase, a Klenow sub-fragment lacking the 3’-5’ exonuclease domain. HoLaMa was overexpressed in *Escherichia coli*, and the enzyme was purified by means of standard chromatographic techniques. High-resolution NMR experiments revealed that HoLaMa is properly folded at pH 8.0 and 20°C. In addition, urea induced a cooperative folding to unfolding transition of HoLaMa, possessing an overall thermodynamic stability and a transition midpoint featuring Δ*G* and *C*_M_ equal to (15.7 ± 1.9) kJ/mol and (3.5 ± 0.6) M, respectively. When the catalytic performances of HoLaMa were compared to those featured by the Klenow enzyme, we did observe a 10-fold lower catalytic efficiency by the HoLaMa enzyme. Surprisingly, HoLaMa and Klenow DNA polymerases possess markedly different sensitivities in competitive inhibition assays performed to test the effect of single dNTPs.

## Introduction

Despite large differences among their catalytic efficiencies, all DNA polymerases known so far share a peculiar molecular architecture, resembling an open right hand [1,2]. The domains of this molecular architecture are accordingly denoted as thumb, palm, and fingers, each one performing a particular function. The binding of a double-stranded DNA (dsDNA) substrate by the enzyme is mainly accomplished by the thumb domain [3], with the fingers domain subsequently binding a deoxynucleoside triphosphate (dNTP) [4] and two Mg^2+^ atoms. First, a Mg^2+^-dNTP complex is bound and paired to the template DNA strand by the fingers domain, and the binding of a second divalent magnesium triggers the so-called fingers closure, i.e. a consistent conformational change leading to DNA elongation [5,6]. The palm domain can indeed catalyse DNA elongation only when the enzyme is in the closed conformation, promoting the nucleophilic attack by the 3’-OH of the primer strand to the α-phosphate of the incoming dNTP [2]. Recently, the requirement of a third divalent cation for the action of DNA polymerases was revealed, suggesting that this third metal ion might promote product formation [7]. Besides this catalytic action, the maintenance of genomes stability demands for a stringent containment of DNA replication errors, and an ensemble of concerted actions contribute to this containment. In particular, it was shown that DNA polymerases catalyse DNA extension featuring exquisite precision, with an estimated frequency of the incorporation of erroneous bases into DNA equal to 10^−4^ [8,9]. Nevertheless, this extraordinary fidelity is not sufficient to guarantee genomes stability, and accessory functions assist DNA polymerases to accomplish this task. DNA elongation can therefore be accompanied by 3’-5’ exonuclease activity, which is responsible for the excision of erroneously incorporated dNTPS. Elegant assays in *Escherichia coli* reported that this 3’-5’ proofreading action lowers the frequency of mismatches down to about 10^−7^ [10]. Remarkably, this frequency can be further reduced to 10^−10^ with the aid of different post-replicative repair systems [11,12].

When proofreading activity is considered, two types of DNA polymerases can be recognized: i) enzymes constituted by a single polypeptide containing both polymerase and 3’-5’ exonuclease activity; ii) DNA polymerases whose elongation and proofreading actions reside in different subunits. The first type is exemplified by the so-called Klenow enzyme, i.e. a fragment of *E*. *coli* DNA polymerase I, obtained by limited proteolysis *in vitro* [13]. A well-known example of the second type is *E*. *coli* DNA polymerase III, the α and ε subunits of which confer to the holoenzyme DNA elongation and proofreading competence, respectively [14–17]. Remarkably, it was reported that α and ε subunits are mutually stimulated [18–20] suggesting that their association favours conformational rearrangements relevant for catalysis. When the Klenow enzyme is considered, one can ask if the polymerase and exonuclease domains can reciprocally affect DNA binding. Interestingly, when this polymerase was crystallized in the presence of a dsDNA containing a C-T mismatch, the single-stranded portion of the template was found to be associated with the exonuclease domain [3]. On the contrary, when the Klentaq enzyme (the Klenow homologue from *Thermus aquaticus*) was co-crystallized in the presence of dsDNA devoid of any mismatch, the substrate did bind to the polymerase domain [21]. These observations suggest an interplay between the polymerase and the proofreading domains of DNA polymerases. To test this, DNA elongation assays were performed with Klenow enzyme, using a dsDNA substrate devoid of mismatches and delaying the addition of dNTPs [22]. In this case, the Klenow enzyme did first catalyse the 3’-5’ reaction with the subsequent addition of dNTPs switching its action to DNA extension [22]. In addition, this switch was determined as intramolecular, i.e. it did not require the dissociation and re-association of the enzyme-DNA complex. Contrary, when dsDNA contains a mismatch, the activity switch was of intermolecular nature, i.e. with the mismatched DNA bound and proofread by the exonuclease domain which then dissociated from the error-free DNA, subsequently bound by the polymerase domain of another enzyme molecule [23]. To hinder the switch between the two Klenow activities, the inactivation of the 3’-5’ exonuclease action by the introduction of two site-specific mutations (D355A, E357A) was previously reported [24]. However, it should be noted that this inactivation does not necessarily abolish the binding of DNA by the proofreading domain of Klenow enzyme. To address this point, we recently constructed a Klenow sub-fragment, i.e. a truncated form of Klenow devoid of the 3’-5’ exonuclease domain [25]. This sub-fragment, denoted HoLaMa, was shown to be competent in catalysing DNA extension, albeit with a lower efficiency when compared to its parental counterpart. In addition, we did also report that the binding of DNA substrates by HoLaMa occurs with similar affinities to those determined for Klenow enzyme [25]. However, it was not investigated neither the intrinsic overall thermodynamic stability of HoLaMa, nor the effects, if any, on enzyme stability triggered by the binding of DNA, so far. Here we report on these points, along with a characterization of the activity of HoLaMa at the expense of different dsDNAs, and we compare the action of HoLaMa with that exerted by the parental Klenow enzyme. Addressing these thermodynamic and catalytic parameters enables us to explain the performance of HoLaMa, in the light of the missing proofreading domain.

## Materials and methods

### Strain, plasmid, growth media, and DNAs

All experiments were performed using *Escherichia coli* TOP10 (genotype: F^-^ *mcrA* Δ(*mrr-hsdRMS-mcrBC*) ϕ80*lacZΔM15* Δ*lacX74 recA1 araD139* Δ(*ara-leu*)*7697 galU galK rpsL endA1 nupG*). For the overexpression of the HoLaMa enzyme, the procedure as previously described was used [26]: briefly, *E*. *coli* TOP10/pBAD-HoLaMa was grown for 9 h at 30°C in LB medium supplemented with 100 μg/mL ampicillin, and the overexpression of the target protein was induced for 15 h time interval, at the same temperature, with 1 mM arabinose. The overexpression of the W866F variant of HoLaMa was induced for 24 h at 15°C, with 1 mM arabinose.

### Purification of HoLaMa wild-type and HoLaMa W866F

The cell pellets containing HoLaMa or the W866F variant were resuspended in buffer A (50 mM Tris-HCl, 50 mM NaCl, 1 mM EDTA, pH 8.0) supplemented with 1 mM phenylmethylsulfonyl fluoride, homogenized with a cold glass potter, and the cell suspension was subjected to sonication (power equal to 18 W, 15 s of impulse, 15 s of cooling interval, total time of 2 min) for 7 cycles. The protein extract was centrifuged (10,000 x g, 20 min), the supernatant was filtered, and the soluble protein extract was immediately loaded onto a Cibacron Blue column (1.6 x 15 cm) previously equilibrated with buffer A. After a washing step performed with buffer A (5 column volumes), HoLaMa was eluted with the same buffer supplemented with 1 M NaCl. The best fractions, according to SDS-PAGE analysis, were pooled, concentrated with an Amicon ultrafiltration cell equipped with a YM-30 membrane (30 kDa cutoff), and then loaded onto a Superdex 200 column (1.6 x 70 cm), equilibrated with buffer B (50 mM sodium phosphate, 50 mM NaCl, 1 mM EDTA, pH 8). The fractions containing less contaminants were directly loaded onto a HiTrap Blue column, equilibrated with buffer B. After washing out the unbound proteins, a linear 0.05–1.2 M NaCl gradient was applied, and HoLaMa was found to elute at about 0.6 M NaCl. The fractions containing pure HoLaMa were pooled, concentrated, and stored at -20°C in buffer B. The purification of HoLaMa W866F was obtained using essentially the same procedure. The concentration of soluble proteins was determined according to Bradford [27].

### Steady-state activity assays

All measurements were performed at 20°C using an Agilent (Santa Clara, CA, USA) Cary 300 spectrophotometer. DNA extension was assayed by using the continuous enzyme-coupled assay as previously described [28]. The release of pyrophosphate by DNA polymerases was detected in 100 mM Tris-HCl buffer (pH 8.0), 5 mM MgCl_2_, 0.25 mM inosine, using inorganic pyrophosphatase, purine nucleoside phosphorylase, and xanthine oxidase (at concentrations of 10, 50, and 500 mU/mL, respectively) as coupling enzymes. The final product generated by these coupling enzymes, i.e. uric acid, was monitored at a wavelength of 293 nm. At this wavelength the extinction coefficient of uric acid was assumed equal to ε = 12.6 x 10^3^ M^-1^ cm^-1^ [29]. All the DNAs used in the present work were synthesized by GenScript (Piscataway, NJ, USA) and by BMR Genomics (Padova, Italy).

### Stopped-flow assay

The conformational rearrangements occurring in HoLaMa W866F upon DNA binding were assayed determining time-resolved tryptophan fluorescence changes. The assay was performed at 20°C, using a KinTek (Snow Shoe, PA, USA) SF2004 stopped-flow equipment. The dead time of the instrument was determined as equal to 2.5 ms. To determine tryptophan fluorescence, excitation wavelength was at 280 nm, and the emission was detected using a longpass filter. The enzyme syringe contained 3.4 μM purified enzyme in 50 mM Tris-HCl, 0.5 mM EDTA (pH 8.0), and the second syringe contained 3.2 μM dsDNA, in 50 mM Tris-HCl, 0.5 mM EDTA, 10 mM MgCl_2_, (pH 8.0). The stopped-flow assays were carried out in the presence of dsDNA obtained by annealing a 40mer template (3’-CGCGCGCGAAAAAAAAAAAAAAAAAAAAAAAAAAAAAAAA-5’) to a 15mer primer (5’-GCGCGCGCTTTTTTT-3’).

### Electrophoresis of DNAs

DNA polymerase reactions (200 μL) were tested in 100 mM Tris-HCl pH 8.0, 5 mM MgCl_2_ containing 1.5 μM of DNA template, 100 μM of each dNTP (dTTP and/or dGTP), and 360 nM HoLaMa. Aliquots of 20 μL were withdrawn from the assay mixture at different time intervals (0, 20, 40, 60, 90, 120 and 180 min), and reactions were stopped by adding 10 mM EDTA. Samples were boiled 5 min in the presence of an Orange loading dye, loaded on TBE-urea gels (15% polyacrylamide containing 7 M urea) and subjected to electrophoresis in TBE at 200 V for 40 min. DNA bands were visualized with ethidium bromide.

### Fluorescence spectroscopy

HoLaMa fluorescence spectra were obtained by exciting the protein tryptophanes at 280 nm, using a Jasco FP-8500 spectrofluorometer. To record the emission spectra (290–400 nm), we used a quartz cuvette and a magnetic stirrer. Samples containing 500 nM HoLaMa (in 50 mM sodium phosphate, 50 mM NaCl, pH 8.0) were incubated for 1 h at 25°C, and the fluorescence of tryptophanes was determined at 329 nm. When urea was used to induce enzyme denaturation, its final concentration in the assays was evaluated by using the refractive index [30], which is related to a solute concentration by:

$$[urea]=117.66*DnD+29.753*{\left(DnD\right)}^{2}+185.56*{\left(DnD\right)}^{3}$$ (1)

where *DnD* represents the difference in the refractive index between buffer containing urea with buffer devoid of urea.

The following equation was used to determine the overall thermodynamic stability, *ΔG* [31,32]:

$$y=\frac{(G_{f}+mf*x)+(Gu+mu*x)*e^{\left(-\frac{\Delta G+mx}{RT}\right)}}{1+e^{\left(-\frac{\Delta G+mx}{RT}\right)}}$$ (2)

where y is the observed value of fluorescence, x the concentration of urea, *G*_f_ and *m*_f_ account for the native as *G*_u_ and *m*_u_ for the unfolding baseline, *m* is cooperativity of unfolding, *R* is the gas constant and *T* the temperature.

Similar fluorescence experiments were performed to determine the dissociation constant, *K*_D_, of HoLaMa-DNA complexes. In this case, the following equation has been used to fit the experimental data [33]:

$$Q=Q_{max}*\frac{A-\sqrt{A^{2}-4n*{[P}_{0}]*[L_{0}]}}{2*{[P}_{0}]}$$ (3)

where *Q* is the quenching of tryptophan fluorescence triggered by DNA binding, *Q*_max_ is maximum of quenching, [P_0_] and [L_0_] are the total protein and the total DNA (ligand) concentration, respectively, and A = *K*_D_ + [P_0_] + *n*[L_0_], with n representing the number of DNA molecules bound per enzyme molecule.

### High-resolution Nuclear magnetic resonance (NMR) spectroscopy

High-resolution NMR spectroscopy was performed using a Bruker Advance III 600 MHz spectrometer equipped with a TCI-H/C/N triple resonance cryoprobe. All experiments were performed at 25°C. Water suppression was achieved by the Watergate sequence. Spectra were processed using TopSpin 2.1 software. One-dimensional proton (1D ^1^H) NMR spectra were recorded both in 50 mM sodium phosphate, 50 mM NaCl (pH 8.0) and in 50 mM Tris-HCl, 50 mM NaCl (pH 8.0). The samples were prepared in a final volume of 500 μL containing 5% (v/v) D_2_O as field lock.

## Results and discussion

### Stability and structural features of HoLaMa

**Fig 1:**
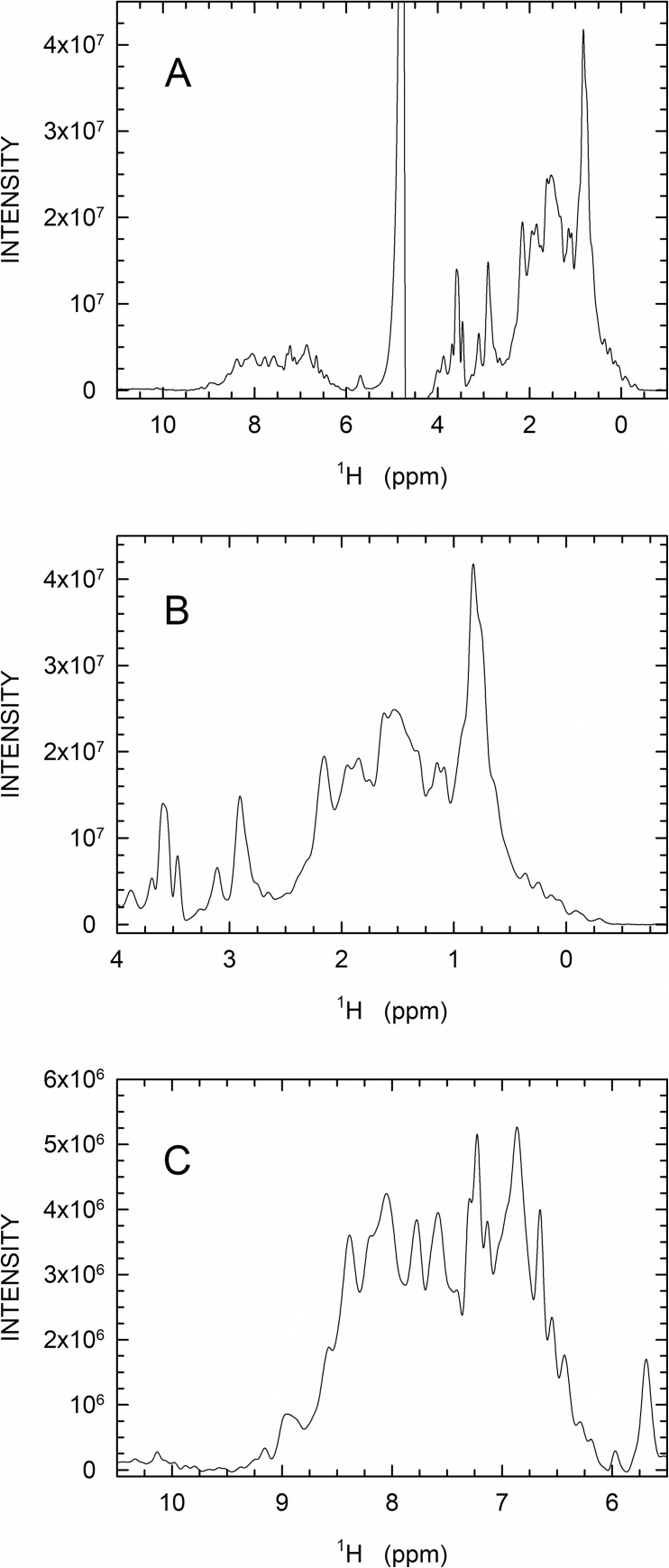
High-resolution one-dimensional proton NMR spectroscopy of HoLaMa. Panel (A): spectrum acquired at 25°C using 50 μM HoLaMa dissolved in 50 mM sodium phosphate buffer containing 50 mM NaCl at pH 8.0. (B) Detail of the aliphatic part of the spectrum. (C) Detail of the aromatic and NH-backbone signal region.

Pioneering attempts to construct Klenow sub-fragments devoid of the 3’-5’ exonuclease domain revealed that the truncation of this domain conferred poor solubility to the majority of the variants designed [34,35]. Accordingly, the production of a properly folded Klenow sub-fragment lacking the proofreading domain requires a consistent re-engineering of the polymerase domain [25,35], and the successful preparation of Klenow sub-fragments whose DNA polymerase activity could be detected has been previously described [25,35]. However, no structural characterization was reported for any of these enzyme variants. Therefore, as a first test, we analyzed HoLaMa by high-resolution 1D ^1^H-NMR spectroscopy (Fig 1). The dispersion seen for the aliphatic (Fig 1A), the aromatic, and the backbone NH signals (Fig 1B and 1C) indicates that HoLaMa is a properly folded protein at the experimental conditions used.

**Fig 2:**
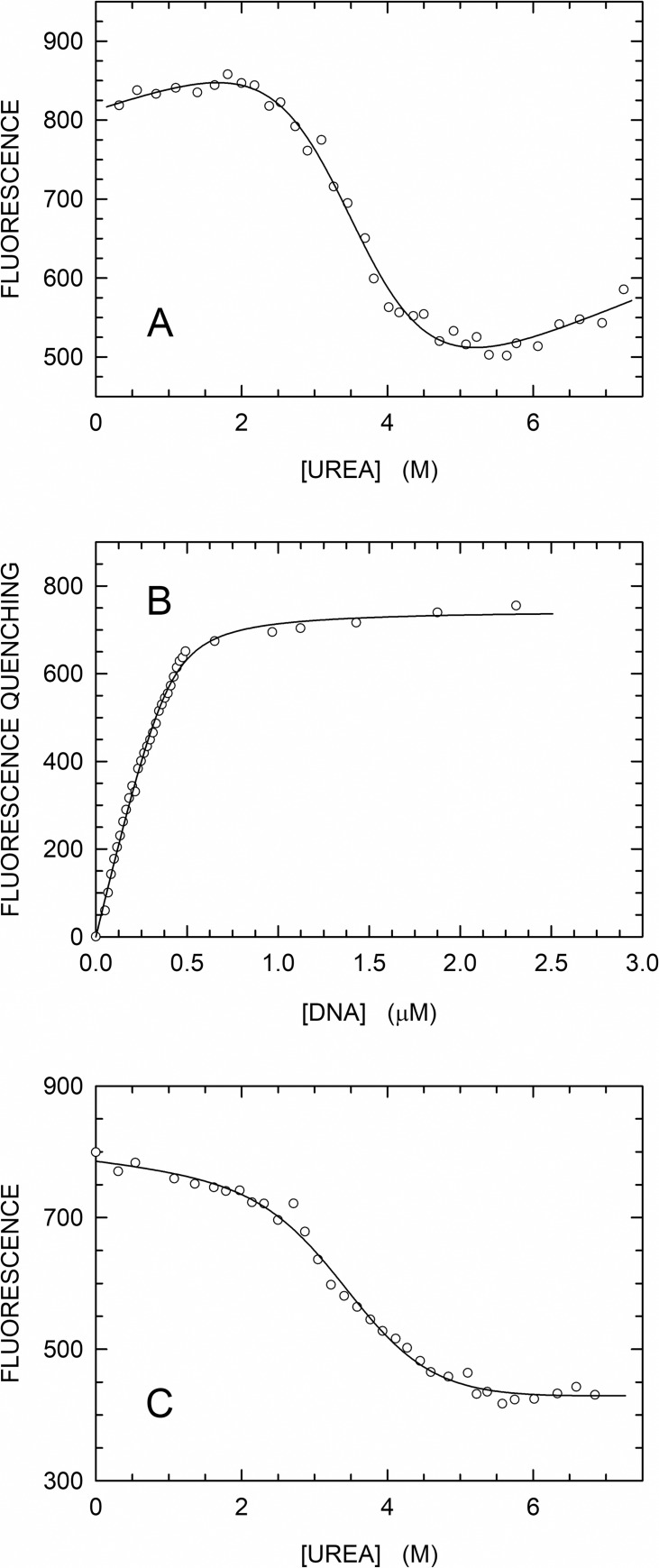
Folding to unfolding transition of HoLaMa as induced by urea. Intrinsic fluorescence of HoLaMa was determined exciting samples at 280 nm and detecting emission at 328 nm. (A) Folded to unfolded transition of 500 nM HoLaMa as induced by the addition of urea. (B) Fluorescence of HoLaMa tryptophanes as affected by binding of the enzyme to the 40mer polyA DNA (see Fig 3). The enzyme concentration was 500 nM. (C) Folded to unfolded transition of HoLaMa bound to the 40mer polyA DNA (see Fig 3) as induced by the addition of urea. Enzyme and DNA concentration were 500 nM each. All the samples were in 50 mM sodium phosphate buffer (pH 8.0), 50 mM NaCl. The continuous lines represent the best fits of the equation described by Pace and Shaw [31] to the experimental data.

To further analyze the overall thermodynamic stability of HoLaMa, we also evaluated its sensitivity to denaturation as induced by increasing concentrations of urea. To this aim, we took advantage of the presence of two tryptophanes in HoLaMa, namely W866 and W924 (coordinates of DNA polymerase I primary structure). The fluorescence of protein tryptophanes is known to be affected by a number of factors [36], among which the polarity of the local environment sensed by this amino acid represents a major determinant. Therefore, the evaluation of protein denaturation by detecting changes in tryptophan fluorescence is a commonly used technique [36]. Accordingly, we analyzed the effect on the fluorescence of the HoLaMa tryptophanes induced by the addition of urea, at constant pH (pH 8.0) and temperature (25°C). First, we recorded the emission spectra of two solutions containing 500 nM HoLaMa, and supplemented or not with 7.6 M urea. Under these conditions, we observed a significant red shift in fluorescence emission upon enzyme denaturation (in agreement with previous observations, [36]), and a maximum value in the difference spectrum at 328 nm (S1 Fig). Consequently, we analyzed subsequent fluorescence experiments at this wavelength (328 nm). When the enzyme fluorescence was determined as a function of urea concentration (Fig 2A), we detected three concentration intervals representative of well-defined protein states [37], namely: i) the folded state, which was observed in the absence or in the presence of urea up to a concentration of 2 M (Fig 2A); ii) the transition from folded to unfolded state, occurring at 2–4 M urea concentration (Fig 2A); iii) the unfolded state, detected at urea concentrations higher than 4 M (Fig 2A). Fitting the equation described by Pace and Shaw [31] to the experimental observations (see Methods), we determined the overall thermodynamic stability of HoLaMa to Δ*G* = (15.7 ± .9) kJ/mol and the cooperativity of unfolding to *m* = (4.5 ± 0.5) kJ/mol*M. Accordingly, the midpoint for the chemical unfolding of HoLaMa is equal to *C*_M_ = (3.5 ± 0.6) M of urea. Remarkably, this value of *C*_M_ is very close to the value determined for the Klenow enzyme, i.e. *C*_M_ = (3.6 ± 0.1) M [38]. However, the thermodynamic stability, *ΔG*, and the cooperativity, *m*, of unfolding for Klenow, i.e. (20.9 ± 1.3) kJ/mol and (6.3±0.4) kJ/mol*M, respectively [38], were significantly different when compared with those for HoLaMa. The higher values for *ΔG* and *m* determined for Klenow enzyme are most likely related to the higher molecular mass of this enzyme in comparison with that of its smaller derivative, HoLaMa. The ratio between the molecular mass of the two enzymes (47 and 68 kDa for HoLaMa and Klenow, respectively) is indeed equal to 0.69, and the ratio between the two *m* values equals 0.71.

**Fig 3:**
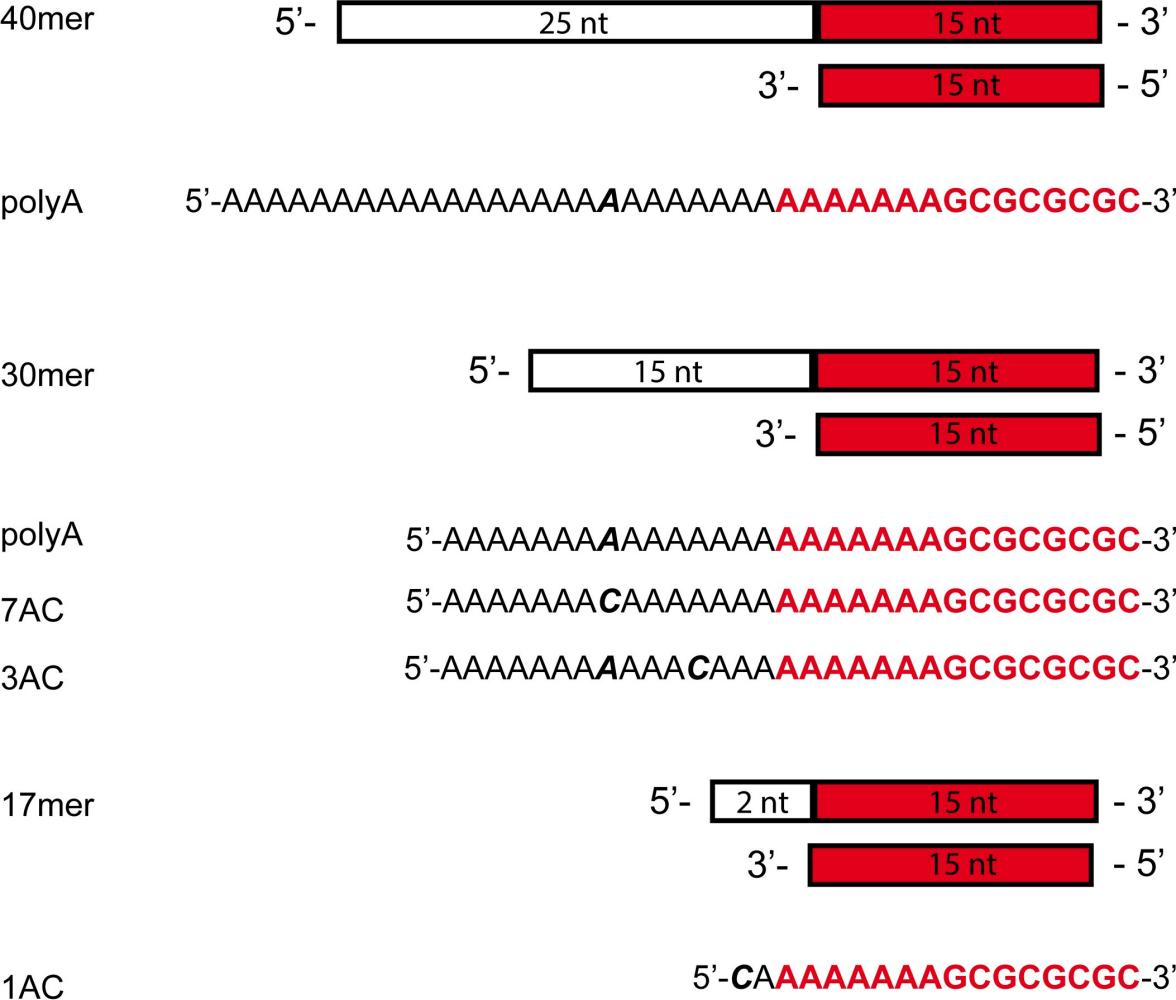
Cartoon of the DNA substrates used in the present work. Cartoon of the dsDNAs used in elongation assays performed in the presence of HoLaMa, Klenow, or Klenow exo^-^. The sequences of the template strands are also reported.

Next, we tested whether the association with DNA does affect or not the thermodynamic parameters diagnostic of HoLaMa stability. First, we excited at 280 nm and detected the emission at 328 nm of samples containing 500 nM HoLaMa and increasing concentrations of the 40mer dsDNA (40mer polyA, Fig 3), previously used to determine the extension activity exerted by HoLaMa [25]. In the presence of DNA/enzyme concentration ratios lower than 1, the fluorescence was found to decrease according to a hyperbolic behavior (Fig 2B). In addition, when the same ratio was higher than 1, the detected fluorescence was not further altered, suggesting that the enzyme was completely saturated with DNA (Fig 2B). This decrease in enzyme fluorescence triggered by the binding to 40mer polyA is in agreement with our previous kinetic assays, performed to detect the fluorescence changes of HoLaMa tryptophanes occurring after DNA binding [26]. Moreover, fitting the equation described by Sachs et al. [33] (see Methods) to the experimental data, we estimated the number of DNA molecules bound to the enzyme, *n*, as *n* = 1.24 ± 0.03, and the dissociation constant, *K*_D_, of the enzyme-DNA complex as equal to *K*_D_ = (35.1 ± 6.1) nM, in reasonable agreement with the 67 ± 19 nM value we previously reported [25].

How does the bound dsDNA affect the overall thermodynamic stability of HoLaMa? Surprisingly, none of the 3 parameters (Δ*G*, *m*, and *C*_M_) describing the folding to unfolding transition of HoLaMa was significantly affected by the binding of dsDNA to the enzyme. Indeed, we determined an overall thermodynamic stability of Δ*G* = (10.9 ± 3.5) kJ/mol, a cooperativity of unfolding of *m* = (3.3 ± 0.9) kJ/mol*M, and a transition midpoint of *C*_M_ = (3.4 ± 1.4) M for the HoLaMa-DNA complex (Fig 2C).

**Fig 4:**
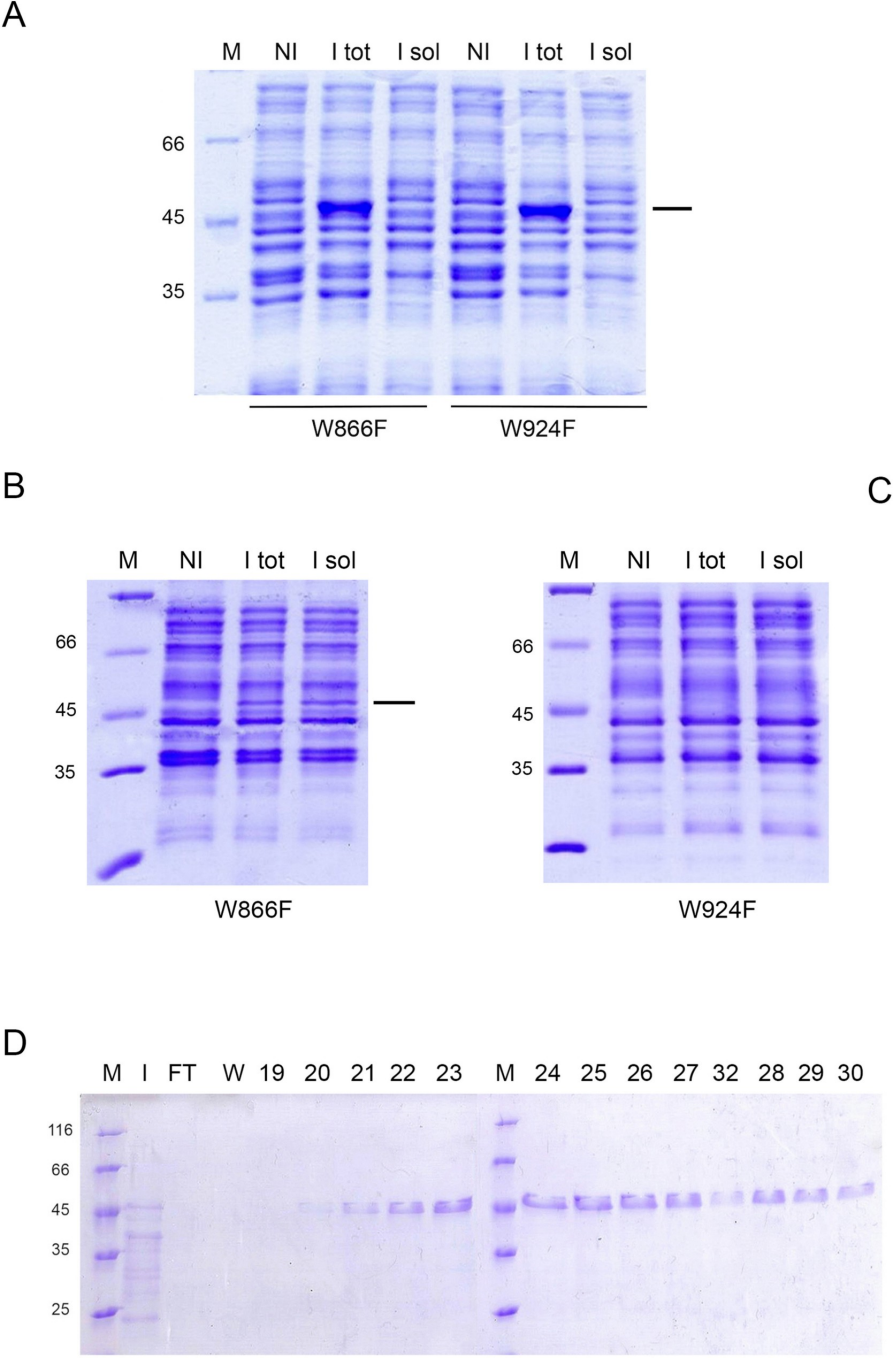
Overexpression and purification of HoLaMa W866F. (A) SDS-PAGE of protein extracts isolated from *E*. *coli* subjected to induction, at 30°C, of HoLaMa W866F or W924F. M, NI, I tot, and I sol indicate: i) molecular mass markers; ii) total proteins extracted from not induced culture; iii) total proteins extracted from induced culture; iv) soluble proteins extracted from induced culture. The molecular masses of the markers are indicated at the left. (B) SDS-PAGE of protein extracts isolated from *E*. *coli* subjected to induction, at 15°C, of HoLaMa W866F. M, NI, I tot, and I sol indicate: i) molecular mass markers; ii) total proteins extracted from not induced culture; iii) total proteins extracted from induced culture; iv) soluble proteins extracted from induced culture. The molecular masses of the markers are indicated at the left. (C) SDS-PAGE of protein extracts isolated from *E*. *coli* subjected to induction, at 15°C, of HoLaMa W924F. M, NI, I tot, and I sol indicate: i) molecular mass markers; ii) total proteins extracted from not induced culture; iii) total proteins extracted from induced culture; iv) soluble proteins extracted from induced culture. The molecular masses of the markers are indicated at the left. (D) SDS-PAGE of fractions eluted from a HiTrap Blue column (5 mL) and containing purified HoLaMa W866F. M, I, and FT indicate molecular mass markers, input, and flow-through, respectively. Fraction numbers and the molecular masses of the markers are indicated at the top and at the left, respectively.

The absence of a significant effect exerted by DNA binding on HoLaMa stability, prompted us to investigate the structural changes linked to enzyme-DNA complex formation. Accordingly, we constructed two HoLaMa site-specific mutants, each bearing a single tryptophan, i.e. W866F and W924F. Unfortunately, we were unable to express the W924F variant in soluble form, neither at 30°C nor at a lower temperature of 15°C (Fig 4). However, when the expression was induced at 15°C, we were able to overexpress HoLaMa W866F in soluble form, although with low yields (Fig 4). We therefore purified this enzyme variant (Fig 4), and we used it to perform stopped-flow assays of DNA binding, under conditions similar to those previously used to probe HoLaMa wild-type containing both tryptophanes.

**Fig 5:**
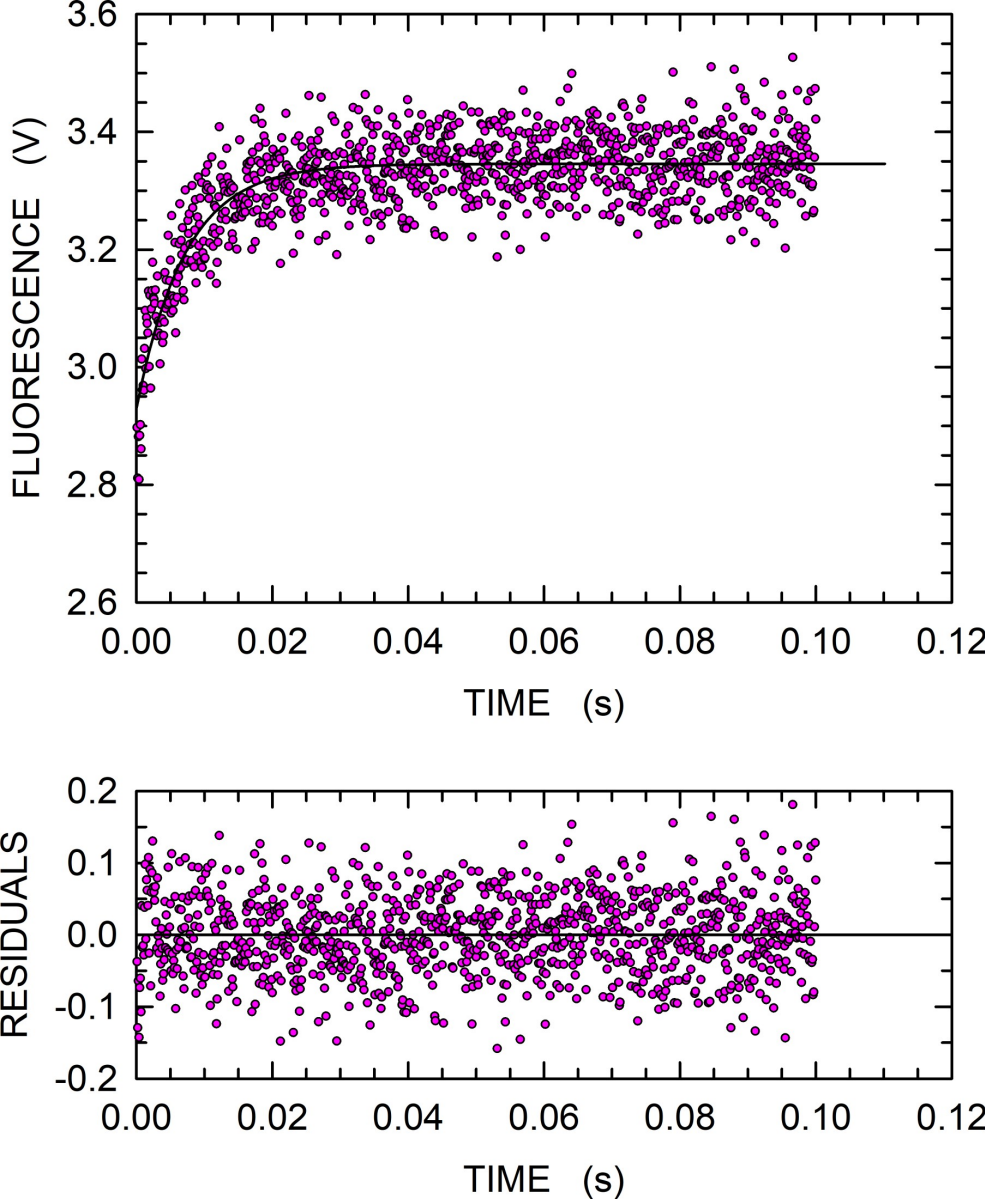
DNA binding by HoLaMa W866F. Stopped-flow assay of HoLaMa W924 fluorescence changes triggered by 3.2 μM 40mer polyA DNA binding, in the presence of 3.4 μM W866F HoLaMa. The continuous line represents the best fit to the experimental observations of a single exponential equation. Residuals are the differences between the observed values and those expected according to the kinetic model used to fit the data.

In particular, we determined the fluorescence of W924 when an excess of enzyme was rapidly mixed to the 40mer polyA DNA (see Methods). The fluorescence of W924 increased upon DNA binding (Fig 5), and the corresponding kinetics can be satisfactorily described by a single-exponential equation, yielding a *k*_*obs*_ equal to (141 ± 7) s^-1^ (Fig 5). This value is about two-fold higher compared to that previously determined for the binding of wild-type HoLaMa to DNA, i.e. *k*_*obs*_ = (64 ± 10) s^-1^ [26]. However, it is important to remark that when the wild-type enzyme was assayed with the same DNA, we observed a fluorescence decrease [26], in agreement with the observations reported here performed in thermodynamic equilibrium (Fig 2B). Accordingly, the conformational rearrangements induced by DNA binding are mainly sensed by W866, and the fluorescence of this tryptophan may therefore be responsible for the signal used here to determine the stability of the HoLaMa-DNA complex (Fig 2C).

### Catalytic action of HolaMa and Klenow at the expense of different substrates

To investigate how the presence of the 3’-5’ domain affects DNA extension activity, we performed DNA elongation assays in the presence of HoLaMa, wild-type Klenow, or the Klenow variant bearing two site-specific substitutions (D355A, E357A) inactivating the proofreading activity (thereafter denoted here Klenow exo^-^). In particular, we used for these assays three different 30mer DNAs featuring a template overhang of 15 bases (Fig 3). It should be mentioned that the enzyme-coupled assay used here monitors DNA extension by means of [28]: i) the action of inorganic pyrophosphatase, which releases orthophosphate from the pyrophosphate generated by DNA polymerases; ii) the phosphorolysis of inosine, and the subsequent oxidation of hypoxanthine to uric acid, in the presence of purine nucleoside phosphorylase and xanthine oxidase; iii) the detection of uric acid at 293 nm. In summary, by using this enzyme-coupled assay one expects to detect two molecules of uric acid (corresponding to the release of two molecules of orthophosphate) for each incoming single nucleotide incorporated into DNA.

**Fig 6:**
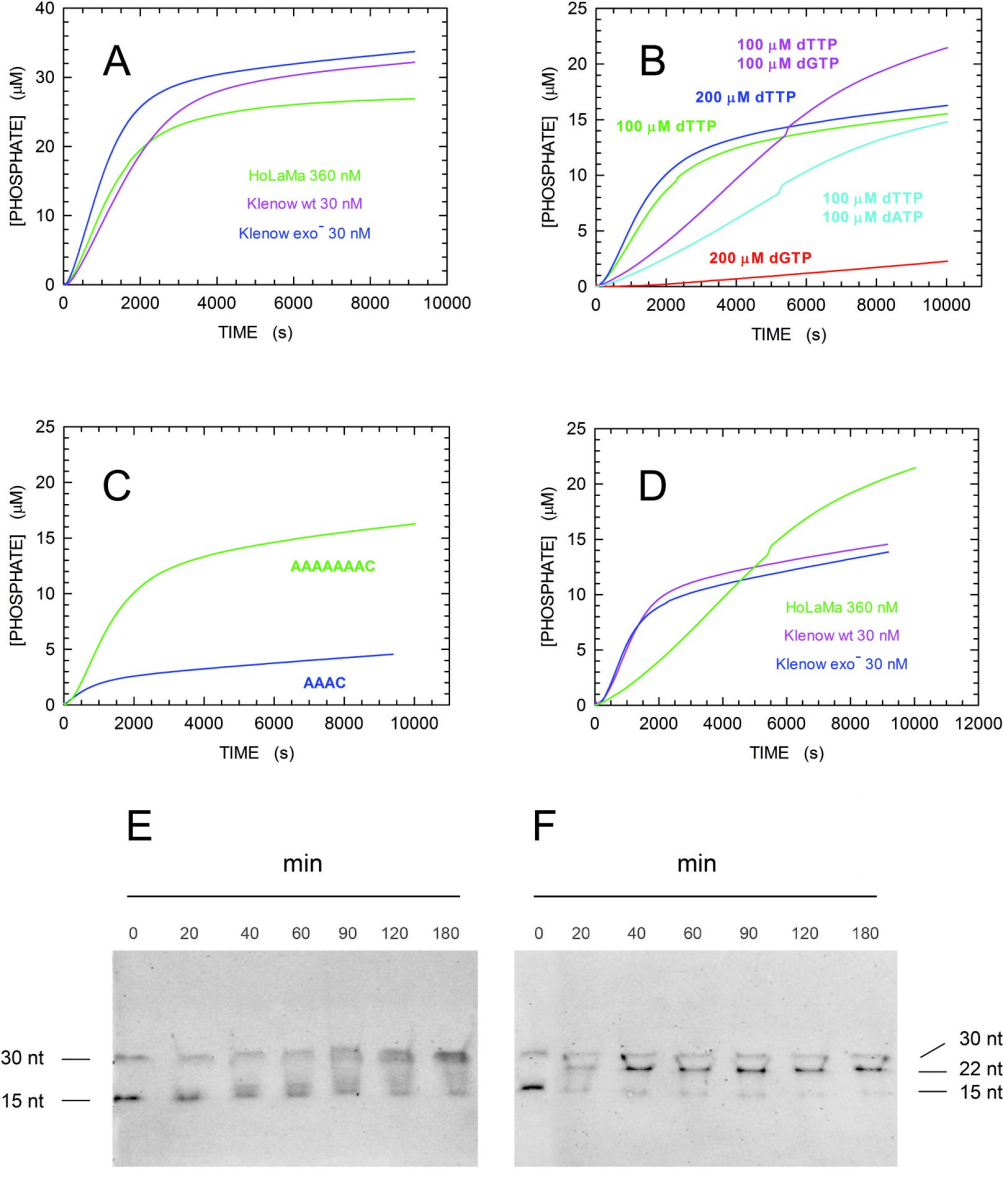
Kinetics of DNA extension catalysed by HoLaMa, Klenow, or Klenow exo^-^ enzyme. (A) Elongation of the 1 μM 30mer polyA DNA in the presence of 100 μM dTTP. The reactions were catalysed by 360 nM HoLaMa (green line), 30 nM Klenow (magenta line) or 30 nM Klenow exo- (blue line). (B) Activity of 360 nM HoLaMa at the expense of 1 μM 30mer 7AC DNA, in the presence of: 100 or 200 μM dTTP (green and blue lines, respectively), dTTP and dGTP 100 μM each (magenta line), dTTP and dATP 100 μM each (cyan line), or 200 μM dGTP (red line). (C) Extension of 30mer 7AC (green line) and 30mer 3AC (blue line) DNA by 360 nM HoLaMa, in the presence of 100 μM dTTP. (D) Kinetics of reactions catalysed by 360 nM HoLaMa (green line), 30 nM Klenow or Klenow exo^-^ (magenta and blue lines, respectively) at the expense of 1 μM 30mer 7AC DNA, in the presence of dTTP and dGTP 100 μM each. The reaction catalysed by HoLaMa is the same shown in (B) and is reported here for comparison. (E, F) Electrophoresis analysis of DNA elongation reactions catalysed by HoLaMa. Electrophoresis of aliquots of reaction mixtures (20 μL) containing 1.5 μM 30mer 7AC DNA (see Fig 3), 360 nM HoLaMa, 100 μM dTTP and 100 μM dGTP (E) or 200 μM dTTP only (F). Both electrophoretic runs were performed using TBE-urea gels (15% polyacrylamide) subjected to constant voltage (200 V) for 40 min.

First, we determined the elongation activity of the three enzymes considered at the expense of the 30mer polyA DNA substrate (Fig 3), by using 1 and 100 μM of DNA and dTTP, respectively. It should be noted that in the presence of the 30mer polyA DNA, dTTP does suffice to fully extend the primer strand (Fig 3), leading to the detection of 30 μM phosphate. Using this setup, the three enzymes yielded similar results, with the extension reaction completed (or almost completed in the case of HoLaMa), in about 30 minutes (Fig 6A). However, it is important to note that HolaMa was used in this assay at a concentration 12-fold higher when compared to Klenow or Klenow exo^-^ (Fig 6A). A similar difference holds when the initial velocity, *v*, of the three reactions is calculated: considering the time intervals yielding zero-order kinetics (linear increase of product concentration as a function of time) we determined *v* as equal to 13.08 ± 0.05, 11.04 ± 0.03, 17.98 ± 0.07 nM/s the activity of HoLaMa, Klenow, and Klenow exo^-^, respectively (Fig 6A). Accordingly, when the enzyme concentrations used in these assays are taken into account, the macroscopic kinetic rate constant, k_obs_, determined for HoLaMa is one order of magnitude lower when compared to those for Klenow or Klenow exo^-^, respectively. Further, we tested the extension by HoLaMa of a 30mer DNA containing a deoxy-Cytosine in the central position of the template overhang (30mer 7AC, Fig 3). Accordingly, using this DNA substrate, the addition of dGTP should be a prerequisite to extend the primer strand beyond the seventh position of the overhang (Fig 3). When 1 μM of DNA was used in the presence of 100 or 200 μM dTTP, the extension did almost stop as expected, i.e. after the generation of 14 μM orthophosphate (Fig 6B, green and blue lines, respectively). It should also be mentioned that the initial velocity in the presence of 200 μM dTTP (*v* = 6.69 ± 0.02 nM/s) was significantly higher when compared to that detected with 100 μM dTTP (*v* = 5.12 ± 0.02 nM/s). When the assay was repeated by using 1 μM 30mer 7AC DNA, 100 μM dTTP, and 100 μM dGTP the extension of the DNA substrate was expected to progress beyond the seventh position (Fig 6B). However, the extension beyond this position proceeded at a slow rate, not obeying zero-order kinetics (Fig 6B, magenta line). These observations obtained by the spectrophotometric enzyme-coupled assay in the presence of the 30mer 7AC DNA (Fig 6B) were additionally confirmed by electrophoresis. When aliquots of a reaction mixture containing HoLaMa, the 30mer 7AC DNA, dTTP, and dGTP were subjected to electrophoresis in polyacrylamide gels, we did observe a full extension of the primer strand in a time scale of two hours (Fig 6E). Contrary, when dGTP was omitted from the reaction mixture, the electrophoretic analysis revealed the presence of an intermediate product, representing partial extension of the primer strand (Fig 6F). Remarkably, the maximal concentration of this intermediate product according to band intensity was generated in a time scale of 40 min, in qualitative agreement with the kinetics observed spectrophotometrically (cf. Fig 6B and 6F). It should be noted that the full extension of the 30mer 7AC primer strand induced by dGTP corresponds to an initial reaction velocity of *v* = (2.963 ± 0.003) nM/s, which is slower when compared to that detected in the presence of dTTP only (Fig 6B). Again, this difference can be appreciated when the progression of the reaction is analyzed electrophoretically. In particular, the decrease of the DNA substrate as a function of time is clearly faster when the assay mixture lacks dGTP (cf. Fig 6E and 6F), suggesting that the correct incorporation of dTTP by HoLaMa is competitively inhibited by dGTP. To further inspect this point, we also analyzed the reaction at the expense of the 30mer 7AC DNA, as catalyzed by HoLaMa in the presence of dTTP and dATP, which is not a prerequisite for full extension of the primer strand. Under these conditions, we observed an incomplete DNA elongation (Fig 6B) and, in addition, a slow initial velocity of *v* = (1.880 ± 0.001) nM/s (Fig 5B). Therefore, it seems reasonable to propose that HoLaMa, when incorporating dTTP, suffers of competitive inhibition by purines. Finally, the reaction in the presence of the same DNA and dGTP only yielded an initial velocity of *v* = (0.270 ± 0.005) nM/s which can be ascribed to the background, by unspecific reactions, of this series of assays (Fig 6B, cyan and red lines, respectively).

We used the 30mer 3AC DNA (Fig 3) to further confirm that the absence of dGTP, which is an inherent prerequisite for full extension of the primer strand, induces the arrest of the elongation reaction. In particular, in the presence of this DNA and of dTTP only, the extension reaction should theoretically progress to the third position of the template overhang, corresponding to the release of 6 μM orthophosphate. Consistently, we did indeed observe that HoLaMa was unable to extend at a considerable reaction velocity the 3AC DNA beyond the second position of the template overhang (Fig 6C).

Finally, we compared the elongation of the 30mer 7AC DNA in the presence of both dTTP and dGTP as catalyzed by HoLaMa, Klenow, or Klenow exo^-^, (Fig 6D). Surprisingly, while HoLaMa was negatively affected by dGTP in this reaction mixture (Fig 6B), both Klenow and Klenow exo^-^ were strongly inhibited when extending the DNA substrate towards (and eventually beyond) the seventh position of the template overhang (Fig 6D). We interpret this inhibition as due to the presence of dTTP, competing with dGTP when this dNTP is necessary for further extension of the primer strand. When the Michaelis-Menten constant, *K*_m_, of Klenow enzyme for dTTP or dGTP was determined, similar values for both dNTPs were obtained [39,40]. In particular, the *K*_m_ value for dTTP was reported as two-fold lower [39] or four-fold higher [40] than the corresponding catalytic constant for dGTP. Accordingly, the excess of dTTP present when the extension of the 30mer 7AC DNA calls for dGTP could be responsible for the inhibition of Klenow polymerase observed here (Fig 6D).

**Fig 7:**
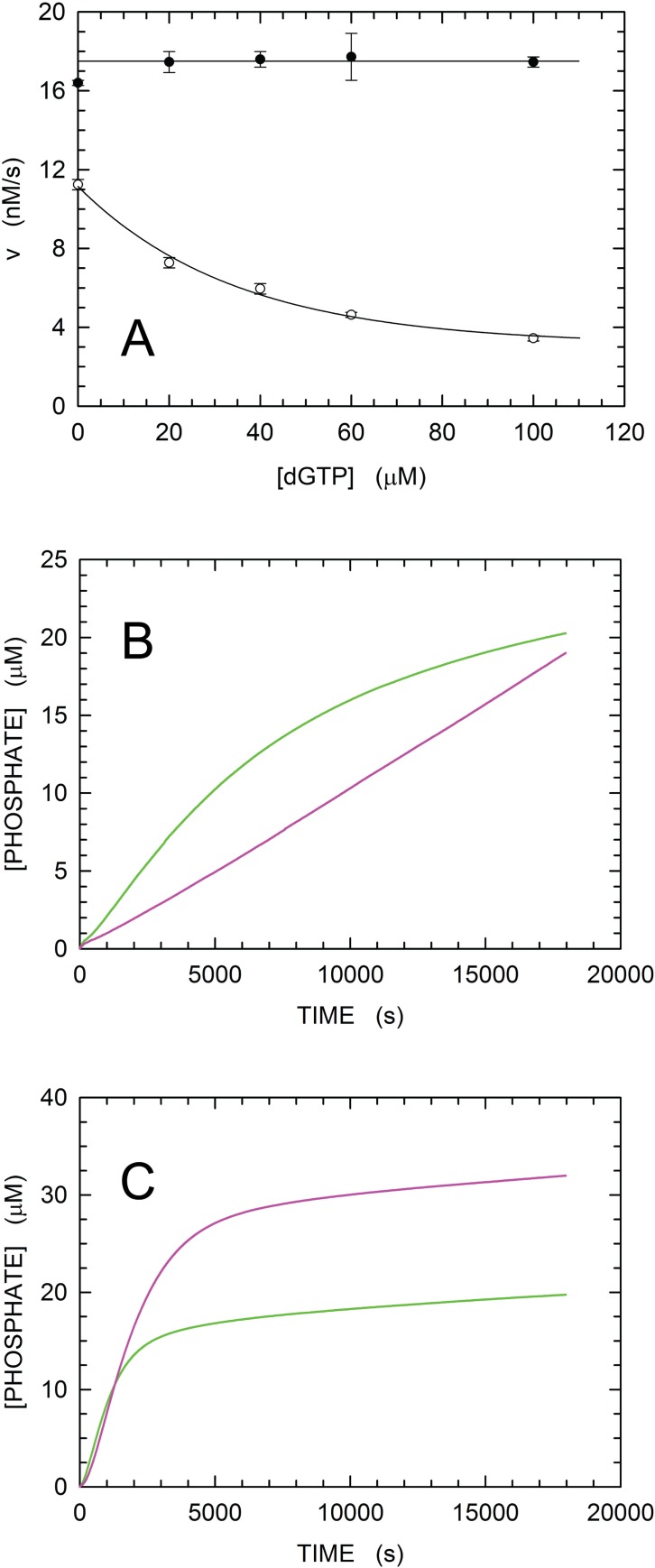
Sensitivity of HoLaMa and Klenow enzymes to the competitive inhibition exerted by dGTP. (A) Assay mixtures containing 360 nM HoLaMa (empty circles) or 30 nM Klenow exo^-^ (filled circels) in 100 mM Tris (pH 8.0), 5 mM MgCl_2_, 0.25 mM inosine, were used to test the extension of 1 μM 30mer polyA DNA. Reactions were started by the addition of 100 μM dTTP, and the pyrophosphate released by the polymerases was determined using a previously described enzyme-coupled assay [28]. The competitive inhibition, if any, triggered by dGTP on the action of both HoLaMa and Klenow exo^-^ was evaluated at concentrations ranging from 20 to 100 μM of dGTP. (B) Activity of 360 nM HoLaMa at the expense of 10 μM 17mer 1AC DNA (see Fig 3), in the presence of: 100 μM dTTP (green line), or dTTP and dGTP 100 μM each (magenta line). (C) Activity of 30 nM Klenow exo^-^ at the expense of 10 μM 17mer 1AC DNA, in the presence of: 100 μM dTTP (green line), or dTTP and dGTP 100 μM each (magenta line).

To investigate this point more in detail, we analyzed the elongation of the 30mer polyA DNA (which does not require dGTP for its full extension, see Fig 3) in the presence of 100 μM dTTP and variable concentrations of dGTP, ranging from 20 to 100 μM. Under these conditions, we compared the initial velocities of the reactions catalyzed by 360 nM HoLaMa or 30 nM Klenow exo^-^. Surprisingly, we observed a sharp difference between the actions of these two enzymes. Klenow was indeed completely insensitive to the competitive inhibition by dGTP, but the activity of HoLaMa was strongly inhibited by this dNTP (Fig 7A). In particular, when dTTP and dGTP were present at equimolar concentration, HoLaMa did perform at a level of 30% when compared to the efficiency detected in the absence of the competitive inhibitor, i.e. dGTP (Fig 7A). We performed further assays to ascertain whether or not the inhibition exerted by dGTP on HoLaMa is dependent on the structure of the DNA substrate. To this aim, we used a short dsDNA containing a 17mer template and denoted 1AC (Fig 3). This dsDNA substrate has an overhang consisting of two nucleotides, calling for dTTP and dGTP at the proximal and distal positions, respectively (Fig 3). When 10 μM of this substrate was used to assay HoLaMa activity, in the presence of dTTP and dGTP (100 μM each) or 100 μM dTTP only, we observed a significant inhibition triggered by dGTP (Fig 7B). The activity was indeed determined as equal to 2.200 ± 0.007 nM/s in the presence of dTTP only and to 1.100 ± 0.006 nM/s when both dTTP and dGTP were added to the reaction mixture (Fig 7B). Moreover, HoLaMa extended the 1AC DNA with the lowest efficiency among those exerted at the expense of the different DNA substrates (cf. Fig 7B with Fig 6A, 6B and 6C). Contrary to HoLaMa, and as expected, Klenow exo^-^ was not significantly affected by dGTP when extending the 1AC DNA. The activity detected at the expense of this DNA substrate was indeed equal to 9.80 ± 0.04 and to 9.60 ± 0.05 nM/s in the presence of dTTP only or in the presence of dTTP and dGTP, respectively (Fig 7C).

The sensitivity of HoLaMa towards the competitive inhibition by dGTP (Figs 6B and 7) and by dATP (Fig 6B) is similar to that reported for the *Thermus thermophilus* DNA polymerase [41,42]. The incorporation of dTTP by this enzyme was shown to be moderately and strongly inhibited by dGTP and dATP, respectively [41,42]. However, it has to be noted that: i) the different sensitivity to competitive inhibition by Klenow and HoLaMa DNA polymerases, as shown here, is linked to the deletion from Klenow of the proofreading domain which is lacking in HoLaMa; ii) the simple disruption of the proofreading activity does not seem to significantly alter the catalytic properties of the Klenow enzyme in the presence of equimolar concentrations of dTTP and dGTP (Fig 6D). Therefore, we propose that the competitive inhibition suffered by HoLaMa resides in structural properties which are not related to the proofreading activity but linked to other functions of the deleted 3’-5’ exo domain, which is known to be competent in dsDNA melting [43], and in binding ssDNA [3,43,44] and dNMPs [44]. The association of Klenow DNA polymerase to homopolymeric substrates occurs with the following order of decreasing affinity: polydA ~ polydG > polydT > polydC [45]. Remarkably, Klenow enzyme does not feature a pronounced selectivity towards the mere binding of dNTPs [46]. However, the binding of a particular dNTP to the enzyme associated to DNA affects the dissociation constant of the resulting complex. Indeed, when a dsDNA bound to Klenow was calling for dCTP to obtain a correct pairing, the *K*_D_ for the DNA was 0.1 nM in the presence of dCTP [47]. However, *K*_D_ values equal to 22, 9, and 1.6 nM were determined in the presence of dGTP, dATP, and dTTP, respectively [47]. According to these observations [47], and to the findings reported for the affinity towards homopolymeric DNAs [45], the polymerase site of Klenow features higher affinity for purines than for pyrimidines. Therefore, we propose that this characteristic of Klenow enzyme translates into competitive inhibition exerted on HoLaMa by dGTP (Fig 7) and by dATP (Fig 6B). Moreover, it will be of interest to investigate the mechanism underlying the competitive inhibition reported here, e.g. assessing whether the proofreading domain, independently of its activity, accelerates or not any step required to prevent inhibition by dNTPs unnecessary for an elongation reaction. In this context, fingers closure assays seem appropriate to investigate this point quite in detail.

Biotechnologically speaking, proofreading defective DNA polymerases may represent ideal enzymes to introduce random mutations in genes coding for enzymes to which directed evolution is applied [48–49]. At present, this is accomplished using Taq DNA polymerase to perform mutagenic PCR, a DNA amplification procedure devised to increase the frequency of replication errors by supplementing the reaction mixture with manganese, and with non-equimolar dNTPs [50]. However, these experimental conditions feature the drawback of lowering the yield of DNA amplification [50]. Disposing of DNA polymerases featuring intrinsic low replication fidelity would therefore be extremely useful for the directed evolution of enzymes. With the construction of HoLaMa we have shown that it is indeed possible to engineer an enzyme to produce an artificial DNA polymerase lacking both 5’-3’ and 3’-5’ exonuclease domains. Accordingly, engineering of a single-domain, thermostable, and error-prone DNA polymerase might represent an appealing challenge for future work.

In summary, it is our hope that the availability of HoLaMa and the biophysical properties as reported here will contribute to a better understanding of the general mechanisms underlying competitive inhibition in DNA polymerases.

## Conclusions

We recently reported on the construction of the HoLaMa DNA polymerase, a Klenow sub-fragment lacking the 3’-5’ exonuclease domain [25]. This enzyme was obtained by designing a synthetic gene coding for the polymerase domain of Klenow and containing eleven mutations, the majority of which were necessary to stabilize and to confer appropriate solubility to the artificial HoLaMa DNA polymerase. Here, we show that this engineered DNA polymerase features a substantial overall thermodynamic stability, comparable to that which has been previously reported by others for the Klenow enzyme possessing the 3’-5’ exonuclease domain. In addition, we did surprisingly observe that the deletion of the proofreading domain in HoLaMa alters the sensitivity of Klenow to the competitive inhibition exerted by dNTPS. HoLaMa and Klenow responded in markedly different ways when exposed to dNTPs unnecessary, or necessary at low concentrations, for the extension of a DNA primer strand. Overall, the thermodynamic stability and the peculiar catalytic features of HoLaMa suggest that this enzyme represents a promising tool for DNA manipulation.

## Supporting information

S1 Fig Fluorescence emission spectra of native and denatured HoLaMa. (A) Solutions containing 500 nM HoLaMa (in 50 mM sodium phosphate, 50 mM NaCl, pH 8.0) and supplemented (blue line) or not (red line) with 7.6 M urea were excited at 280 nm. The emission spectra were recorded over the 290–400 nm interval and are reported in arbitrary units (a.u.). (B) Difference emission spectrum between native and denatured HoLaMa.
(TIF)
