# Supplementary figures and images for: Structural and catalytic insights into HoLaMa, a derivative of Klenow DNA polymerase lacking the proofreading domain
Source: PLoS One. 2019 Apr 10;14(4):e0215411. doi: 10.1371/journal.pone.0215411 (PMC6457538; doi:10.1371/journal.pone.0215411)

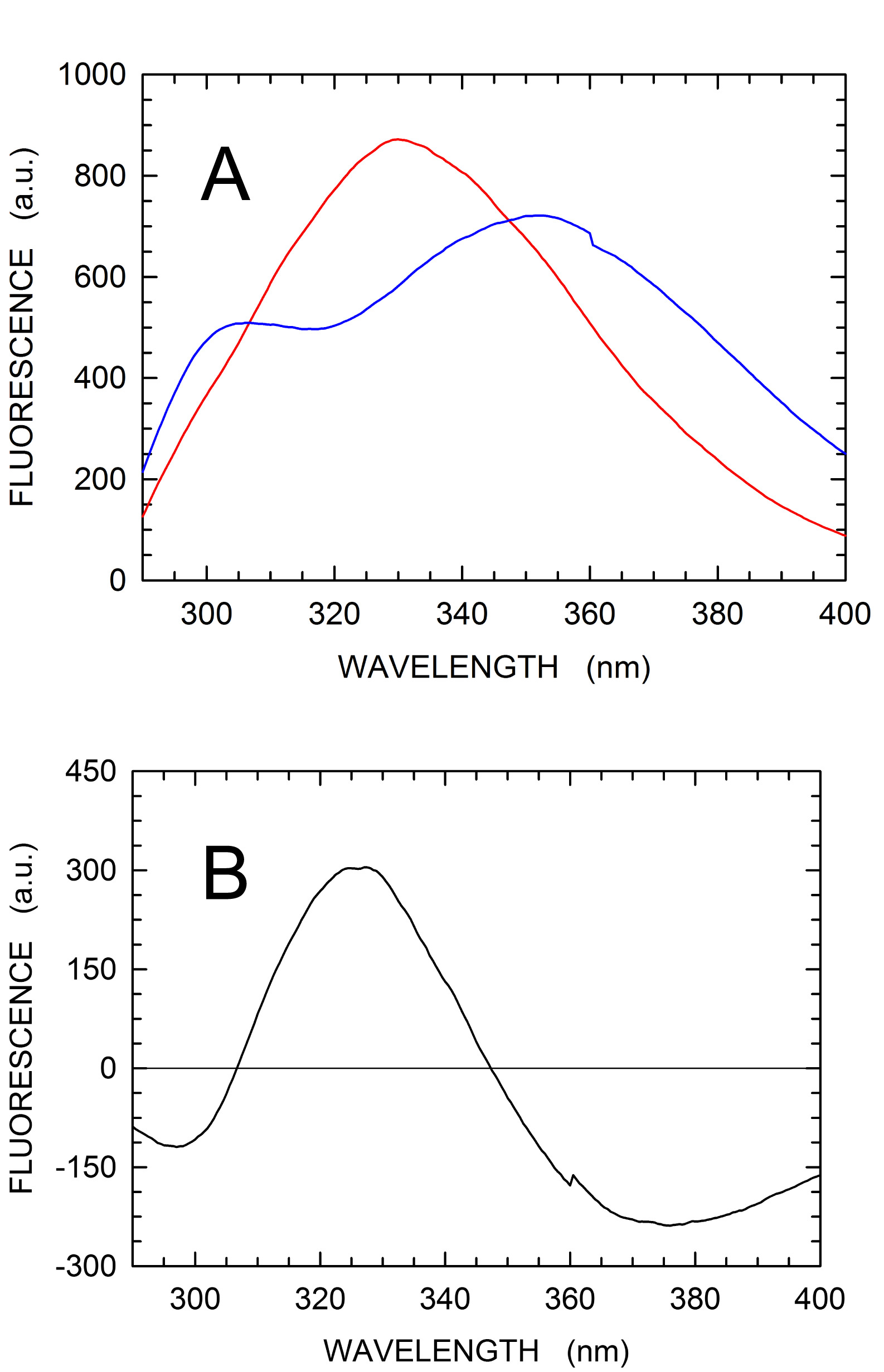

Supplement: S1 Fig — (A) Solutions containing 500 nM HoLaMa (in 50 mM sodium phosphate, 50 mM NaCl, pH 8.0) and supplemented (blue line) or not (red line) with 7.6 M urea were excited at 280 nm. The emission spectra were recorded over the 290–400 nm interval and are reported in arbitrary units (a.u.). (B) Difference emission spectrum between native and denatured HoLaMa. (TIF) [file pone.0215411.s001.tif]
